# mRNA vaccine-induced antibodies more effective than natural immunity in neutralizing SARS-CoV-2 and its high affinity variants

**DOI:** 10.1038/s41598-022-06629-2

**Published:** 2022-02-16

**Authors:** Yunkai Yu, Dominic Esposito, Zhigang Kang, Jianming Lu, Alan T. Remaley, Valeria De Giorgi, Leonard N. Chen, Kamille West, Liang Cao

**Affiliations:** 1 Genetics Branch, Center for Cancer Research, National Cancer Institute, 37 Convent Dr. MSC 4265, Bldg 37, Rm 6040, Bethesda, MD 20892 USA; 2 Protein Expression Laboratory, NCI RAS Initiative, Frederick National Laboratory for Cancer Research, Frederick, MD USA; 3 Codex BioSolutions, Inc, Gaithersburg, MD USA; 4 Lipoprotein Metabolism Section, National Heat, Lung, and Blood Institute, Bethesda, MD USA; 5 Department of Transfusion Medicine, National Institutes of Health Clinical Center, Bethesda, MD USA

**Keywords:** Vaccines, Virology, Infectious diseases, Viral infection, Microbiology, Diseases, Biomarkers, Predictive markers, Translational research, Medical research, Biomarkers, Predictive markers

## Abstract

Several variants of SARS-CoV-2 have emerged. Those with mutations in the angiotensin-converting enzyme (ACE2) receptor binding domain (RBD) are associated with increased transmission and severity. In this study, we developed both antibody quantification and functional neutralization assays. Analyses of both COVID-19 convalescent and diagnostic cohorts strongly support the use of RBD antibody levels as an excellent surrogate to biochemical neutralization activities. Data further revealed that the samples from mRNA vaccinated individuals had a median of 17 times higher RBD antibody levels and a similar degree of increased neutralization activities against RBD-ACE2 binding than those from natural infections. Our data showed that N501Y RBD had fivefold higher ACE2 binding than the original variant. While some antisera from naturally infected subjects had substantially reduced neutralization ability against N501Y RBD, all blood samples from vaccinated individuals were highly effective in neutralizing it. Thus, our data indicates that mRNA vaccination may generate more neutralizing RBD antibodies than natural immunity. It further suggests a potential need to maintain high RBD antibody levels to control the more infectious SARS-CoV-2 variants.

## Introduction

Humoral immunity against SARS-CoV-2 can be achieved with either natural infection or vaccination. Most people who had COVID-19 developed sustained serological responses^1–4^. While the antibodies can be detected in most SARS-CoV-2 infected individuals, the antibody levels are highly variable^5^. On the other hand, some of the current assays have limitations in detecting either circulating antibodies^5^ or neutralizing activities^2–4^ in those individuals. In one instance, those with IgG antibody results were semi-quantitative and those with titers of less than 1:320 would fail to produce detectable neutralizing activities^3^. There are many studies reporting reinfection by SARS-CoV-2^6–9^, whereas in some of the studies, the neutralizing antibodies were shown to have a protective role^10, 11^. In one recent report, individuals who were SARS-CoV-2 seropositive from prior exposure had an estimated 80% reduction of subsequent risk for reinfection^12^. Several late-stage clinical studies demonstrated the effectiveness of COVID-19 vaccines^13–17^. The mRNA-based vaccines have achieved remarkable clinical efficacy in protecting the vaccinated subjects against COVID-19^13–15^. A previous phase 1 study showed that neutralizing activity elicited by Moderna’s mRNA vaccine was in the upper half of that of convalescent plasma specimens^18^. There are a few urgent issues to be addressed. First, it is important to understand the differences in specific antibodies and neutralization activities between the vaccine acquired and natural immunity against SARS-CoV-2 infections. This information would help to determine the need to vaccinate against SARS-CoV-2 in those with natural immunity, especially against the variants. Second, as there are many SARS-CoV-2 vaccines already administered to the general population, it is important to determine and monitor antibody levels and neutralization activities to estimate the durations of protection. While clinical trial outcome is the gold standard for efficacy, these vaccine trials take a long time to execute and are subject to extensive variations, especially with emerging and different SARS-CoV-2 variants, which make comparisons difficult. Thus, effective surrogates would be helpful for their assessment. Third, due to emerging SARS-CoV-2 variants, many with increased affinity to the cell surface ACE2 receptor, it is important to adapt the vaccination strategy accordingly for optimal protection against COVID-19.

The spike (S) protein of the virus binds to ACE2 through its RBD^19, 20^, which is becoming a key research area for public health due to its roles in developing neutralizing antibodies^21^, and its mutations in emerging SARS-CoV-2 variants spreading rapidly worldwide. The first of such variants with a D614G mutation at the spike protein was shown to increase viral titer and infectivity, yet it was effectively neutralized by antisera^22^. More recently, a UK variant B.1.1.7 was implicated to cause a surge in COVID-19 cases^23^. It had a mutation N501Y in the RBD region that is directly involved in contacting ACE2^24^. N501Y and two other mutations in the RBD domain, K417N/T and E484K, were subsequently founds in SARS-CoV-2 variants from South Africa (B.1.351)^25^ and Brazil (P.1)^26^. The N501Y was of particular interest due to its presence in all three variants and its unique role in mediating a direct contact with ACE2 receptor. Mutational scanning studies of SARS-CoV-2 RBD domain in yeast showed the N501F mutation resulted in several fold increased affinity to ACE2^24^. In a mouse model of SARS-CoV-2, the N501Y mutation emerged and conferred increased affinity towards mouse ACE2 receptor^27^. While several recent studies suggested of vaccine antisera that bind and neutralize this B.1.1.7 variant^28–31^, there is a need to investigate the antibody levels after vaccination and protection against infections. With over 150 million COVID-19 cases worldwide by May 2021, and difference vaccines available, it is also important to understand the differences between vaccination and natural immunity, between vaccines, and to better predict the ability of acquired immunity to protect the subjects against emerging variants. Thus, we chose the original and N501Y RBD that appeared in B.1.1.7 and other SARS-CoV-2 variants for the investigation.

## Results

### mRNA vaccination induces higher anti-RBD antibody levels than natural immunity against SARS-CoV-2

**Figure 1:**
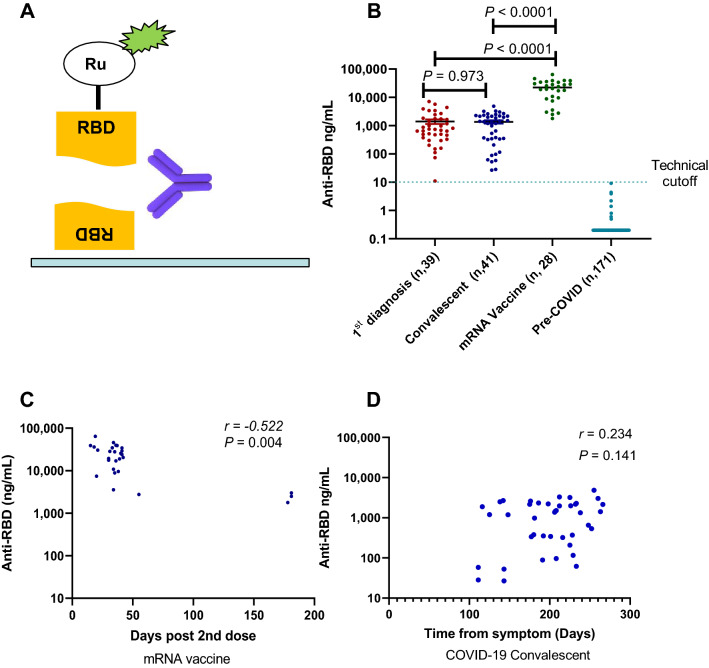
COVID-19 serology assay and antibody levels. (**A**) Diagram of the serology assay using electrochemiluminescence detection method. The capture RBD protein was attached to the assay plate via biotin and the detection RBD was conjugated to the voltage-sensitive ruthenium (Ru) molecule to emit light. (**B**) Quantitative RBD antibody data from sera samples of three cohorts: (1) samples on the first seropositive test, (2) convalescent donors with documented COVID-19, (3) donors without prior COVID-19 and completed two doses of mRNA vaccines. Negative control samples were collected from healthy donors prior to Jan. 2020. (**C**) The RBD antibody levels and time association in vaccinated donors (*r* = − 0.522, *P* = 0.0044). (**D**) Lack of RBD antibody levels and time association in COVID-19 convalescent donors (*r* = 0.234, *P* = 0.141).

Many of the current serology tests for RBD have limitations on sensitivity, dynamic range, and unprecise or semi-quantitative^3, 32, 33^. To accurately determine the levels of anti-RBD antibody in SARS-CoV-2 infected individuals, we developed an electrochemiluminescence-based serology assay for exceptional sensitivity and dynamic range. The assay is based on two antibody-antigen interactions for maximum specificity (Fig. 1A). We purified recombinant wildtype (WT) RBD from transiently transfected 293 cells and labeled it with either biotin for antibody capture or ruthenium (Ru)-tag for antibody detection^33^. Two commercially available RBD monoclonal antibodies (Mab), Mab D001 and D003, were randomly selected (Sino Biological, Wayne, PA) and used as the calibrator (D003) and the reference standard (D001) for accurate quantification. Linear quantification range of at least 1,000-fold was achieved (Supplementary Fig. 1A). The assay was subsequently simplified from a three-step to a single incubation step without the loss of performance (Supplementary Fig. 1B), which would allow the rapid testing completed within one hour.

The RBD serology assay was validated. In five consecutive tests, the average lower limit of quantification (LLoQ) was 0.57 ng/mL and the max LLoQ value of 0.97 ng/mL, with a linear quantification range of 1–1000 ng/mL (Supplementary Fig. 2A). Thus, 1 ng/mL was set as the LLoQ. When serum samples were adjusted for 1:10 dilution, the assay had a testing quantification range of 10–10,000 ng/mL (Supplementary Fig. 2A). In two sets of repeats of five experiments, the precision of quantification was 15.3 and 15.9%. The accuracy of the analysis was good with 100% spike recovery in two experiments, and intra-day CVs of 2.2 and 2.0%. Thus, the RBD serology assay has an LLoQ of 1 ng/mL and acceptable precision and accuracy.

The clinical validation study of the test was performed using 41 serum samples from 33 convalescent donors (Supplementary Table 1) with documented history of COVID-19 prior to September 1, 2020, months prior to the first reported alpha variant which contains RBD mutation N501Y and from 171 healthy donors collected before January 2020. None of the convalescent donors received COVID vaccines. The result revealed all negative samples were below the LLoQ (N = 171), whereas all convalescent donors were positive in the test (N = 41; Fig. 1B). The median RBD antibody level is 1.33 µg/mL with a large 170-fold range for RBD antibody levels in this small group of convalescent samples (range 27–4800 ng/mL; Fig. 1B). The RBD antibody assay has an area under curve (AUC) in Receiver Operator Characteristic (ROC) analysis of 1.00 (Supplementary Fig. 2B) with this groups of 41 samples from those with previously confirmed COVID-19 infections, thus, giving it an 100% sensitivity. It further has 100% specificity due to a lack of any quantifiable antibody levels in serum samples from pre-COVID-19 donors (N, 171).

In comparation with a commercially available spike S1 protein antibody test from Ortho Diagnostics, our RBD antibody test showed strong correlation with it (*r* = 0.679, *P* < 0.0001) (Supplementary Fig. 3). However, our assay exhibited a greater linear dynamic range at lower concentrations and improved sensitivity (41/41) over Ortho’s serology test (39/41) (Supplementary Fig. 3). Thus, our RBD antibody test is quantitative, and has exceptional sensitivity and a large dynamic range.

### Clinical evaluation of RBD antibody levels in mRNA vaccinated and naturally infected individuals

We further evaluated the test in blood samples taken from those who went to the National Institutes of Health (NIH) Clinical Center for COVID-19 tests and were SARS-CoV-2 serology test positive for the first time. Similar levels of RBD antibody were detected in this newly diagnosed group (median, 0.74 µg/mL), which is not significantly different from those of convalescent individuals (*P* = 0.973) (N = 39, Fig. 1B). Interestingly, the blood from donors who completed two doses of mRNA vaccines (Pfizer or Moderna, N = 28) had much higher RBD antibody levels than that of the convalescent group and the newly diagnosed group (Fig. 1B, P < 0.0001). The median level of RBD antibody for the mRNA vaccine group was 22.3 µg/mL, which was 16.8-fold and 30.1-fold higher than that of the convalescent and newly diagnosed groups.

There were differences in age and in sampling time between the convalescent and immunized groups. The immunized group had a median age of 35.5 years which was different from that of the convalescent group at 59.0 (*P* < 0.0001) (Supplementary Table 1). However, the 17-fold differences in antibody levels between vaccinations and infections could not be explain by age alone, as there was an age overlap between the groups, and there was no age and antibody level association for both convalescent (*P* = 0.293) and immunized groups (*P* = 0.361) (Supplementary Fig. 4).

We examined the RBD antibody levels of the vaccine group and time association and noticed a correlation (*r* = − 0.522, *P* = 0.004; Fig. 1C). There was a difference in the antibody levels from samples taken within 2 months and at 6 months post second dose where the 6 months antibody levels were sharply lower (*P* = 0.001; Supplementary Fig. 5). In contrast, convalescent sera did not exhibit a correlative between time and antibody levels, with a median follow-up time of 207 days from the disease onset (*r* = 0.234, *P* = 0.141; Fig. 1D). The analysis of paired samples from same individuals in the convalescent group showed no change in antibody levels at two different time points (*P* = 0.396), whereas the paring was highly effective (*r* = 0.912, *P* = 0.0007; Supplementary Fig. 6). Hence, the data suggests that the antibody levels of convalescent sera did not decline significantly for 8 months post infections, whereas the ultrahigh RBD antibody levels achieved with mRNA vaccines could be subject to a more rapid decline.

### Anti-RBD antibody concentration-dependent neutralization against RBD-ACE2 binding with COVID-19 antisera from natural immunity

**Figure 2:**
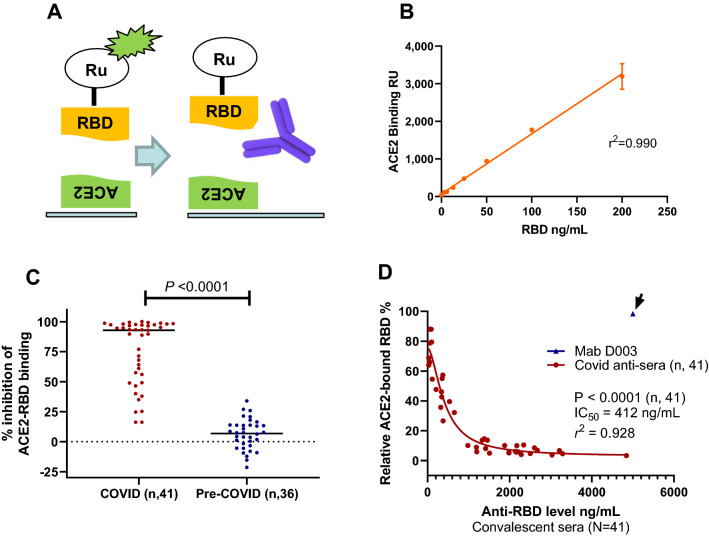
Neutralization assay, sensitivity, specificity, stability, and association with RBD antibody levels in COVID-19 convalescent sera. (**A**) Diagram of RBD and ACE2 biding assay and serum neutralization assay, where the neutralizing antibody prevents RBD from binding to ACE2. (**B**) Linearity of the RBD-ACE2 binding assay where increased RBD results in linear increase of binding signal (*r*^2^ = 0.990). (**C**) Neutralization data showing the convalescent sera have much higher neutralizing ability (*P* < 0.0001). (**D**) Serum neutralization assay results using COVID-19 convalescent sera showing strong correlation between anti-RBD levels and neutralizing activity (*P* < 0.0001, N = 41). The inhibitory concentration (IC_50_) shown was determined using nonlinear regression inhibitory model using RBD antibody levels in the sera, without accounting for a sixfold sample dilution.

To accurately quantify the ability of the antisera in neutralizing RBD and ACE2 binding, we developed an electrochemiluminescence based protein binding assay using recombinant RBD and ACE2 proteins as illustrated in Fig. 2A. When RBD was added to the assay wells, there was an excellent linear relationship between added free RBD and the luminescence signal from the RBD bound to ACE2 (*r*^2^ = 0.99; Fig. 2B). Therefore, the ACE2 binding assay provides a precise quantification of free RBD capable of binding to ACE2. The neutralization assay had an analytical precision of 6.8% (inter-day CV value, N = 3) using Mab D001 as the reference for neutralization assay. To clinically validate the sensitivity and specificity of the RBD-ACE2 binding assay, we analyzed the percentage of inhibition with the 41 COVID-19 convalescent sera and a comparable number of pre-COVID-19 control sera. The results showed that the 41 COVID-19 sera had a significantly higher inhibitory effect against RBD-ACE2 binding (*P* < 0.0001; Fig. 2C). The median levels of inhibition were 93% for the convalescent sera and 7% for the control sera. When comparing the convalescent sera with the negative controls, the antibody neutralization assay showed high sensitivity and specificity, with an AUC in ROC analysis of 0.986 (Supplementary Fig. 7), indicative of high sensitivity and specificity of the assay. The neutralization assay further has a clinical sensitivity of 90% and specificity of 97%, when a cutoff value (− 30%) was established based on the results of the pre-COVID-19 sera (Mean + 2X SD). Due to some non-specific inhibition of ACE2-RBD binding observed with pre-COVID-19 sera (Fig. 2C), additional work in better refining the cutoff and in determining the accurate sensitivity and specificity is needed. The neutralization assay further demonstrated similar consistency when compared with the RBD antibody test using paired convalescent serum samples taken at different time points, with a correlation coefficient for pairing *r* = 0.952 that was highly significant (*P* = 0.0001; Supplementary Fig. 8). Thus, not only the RBD-ACE2 receptor neutralization assay has excellent sensitivity and specificity for COVID-19 antisera, but also is precise with paired samples.

From the eight donors with paired samples, it is also apparent that those with high anti-RBD levels (> 1000 ng/mL) showed stronger neutralization activity, whereas those with low anti-RBD levels (< 100 ng/mL) showed much lower neutralization activity (Supplementary Fig. 8). The complete analysis of COVID-19 convalescent sera for neutralization activity against RBD-ACE2 binding was performed. The results indicated there was an association between the anti-RBD antibody level and neutralization activity against RBD-ACE2 binding (correlative analysis, *P* < 0.0001; Fig. 2D). There was an extraordinary antibody dose-dependent neutralization activity using the nonlinear regression model with *r*^2^ = 0.928. The dilution adjusted (sixfold) and estimated 50% inhibitory concentration inhibition (IC_50_), based on the analyses of 41 samples, was 69 ng/mL. Five of the 41 convalescent sera had lower than 69 ng/mL RBD antibody, 4 of which were negative in the neutralization assay (< 30% inhibition, Fig. 2C). The neutralization assay was specific, as 5000 ng/mL of the calibrator monoclonal antibody D003, which bound to RBD well but exhibited no neutralization activity against RBD-ACE2 binding (Fig. 2D, data point shown with an arrow). As expected, RBD antibody concentration-dependent inhibition of RBD-ACE binding was confirmed with the second group of diagnostic serum samples (Supplementary Fig. 9). Thus, there is a strong RBD antibody concentration-dependent neutralization activity against RBD-ACE2 binding with two sets of COVID-19 antisera.

### N501Y RBD significantly increased ACE2 binding and attenuated the neutralization ability of COVID-19 convalescent antisera

**Figure 3:**
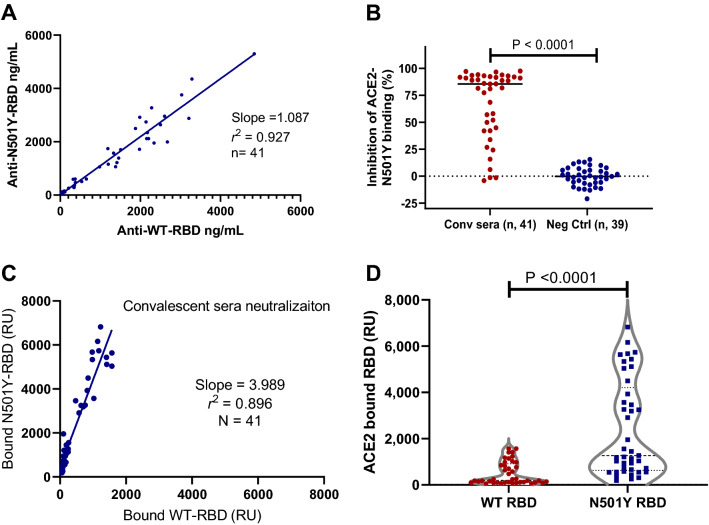
Comparison of antibody levels and neutralization activities against both the wildtype RBD and N501Y RBD proteins with convalescent sera. (**A**) Linearity of the anti-WT-RDB and anti-N501Y-RBD detected in the convalescent sera with a slope of 1.087 (*r*^2^ = 0.927). (**B**) Neutralization data from COVID-19 convalescent sera and negative controls. The convalescent sera had specific neutralizing activity against N501Y-RBD (*P* < 0.0001). (**C**) Linear regression analysis of the ACE2 bound the WT and N501Y RBD detected using the COVID-19 convalescent sera with a slope of 3.99 (*r*^2^ = 0.896, N = 41). (**D**) N501Y RBD have much higher absolute ACE2 binding than the WT RBD in the presence of neutralizing convalescent sera (*P* < 0.0001, N = 41).

To investigate the ability of antisera from convalescent patient to recognize the B.1.1.7 N501Y variant (alpha), we purified N501Y RBD protein and labeled it with the Ru-tag for electro-chemiluminescence assay. We derived a scheme to compare the levels of antibodies against either the WT or N501Y RBD in the same samples (Supplementary Fig. 10). The results with the convalescent donor blood samples showed strong linear correlation between antibodies recognizing both the WT and the N501Y proteins (*r*^2^ = 0.927). Best-fit analysis revealed a slope of 1.09 (95% CI 0.99–1.19; Fig. 3A), suggesting the antisera from COVID-19 convalescent donors bind both the WT and N501Y RBD proteins equally well.

We further analyzed the ability of COVID-19 convalescent antisera to neutralize the binding of the N501Y RBD to ACE2, as in the case of the WT RBD. The results showed the specific neutralization of the antisera from COVID-19 convalescent donors when compared with that of the pre-COVID-19 donor sera samples (*P* < 0.0001; Fig. 3B). The neutralization assay against RBD-N501Y and ACE2 binding was both very sensitive and specific with AUC of ROC analysis of 0.948 (Supplementary Fig. 11). There was further a strong linear correlation between neutralization activity against the WT and the N501Y RBD in ACE2 binding with a slope of 1.03 (*r*^2^ = 0.896, n = 41; Supplementary Fig. 12). Thus, COVID-19 antisera neutralize WT and N501Y RBD with an equal potency.

However, we observed dramatic differences in the ability of the WT and N501Y RBD to bind ACE2. Results from five consecutive experiments showed that N501Y RBD bound to ACE2 at an average of 5.1-fold higher rate than the WT RBD (range 4.1 to 6.1-fold). A representative result is shown (Supplementary Fig. 13). Thus, it can be concluded that N501Y RBD has a much higher affinity to ACE2. We further examined the absolute level of the WT and the N501Y RBD bound to ACE2 in the presence of COVID-19 antisera. While the antisera neutralized both the WT and N501Y RBD at a similar rate, there was four times (slope = 3.99, N = 41) more N501Y RBD bound to ACE2 in the presence of the convalescent antisera (Fig. 3C). This was further confirmed with the COVID-19 diagnostic sera (Supplementary Fig. 14). As more N501Y RBD has a higher affinity to ACE2, there were far more absolute amount of N501Y RBD bound to ACE2 than the WT in the presence of COVID-19 convalescent antisera (*P* < 0.0001, N = 41; Fig. 3D). Thus, natural immunity from the original SARS-CoV-2 infections could not consistently provide sufficient neutralization against N501Y RBD variant from binding to the cellular ACE2 receptor.

### mRNA vaccination results in much more effective neutralization than natural immunity against N501Y RBD from binding to ACE2

**Figure 4:**
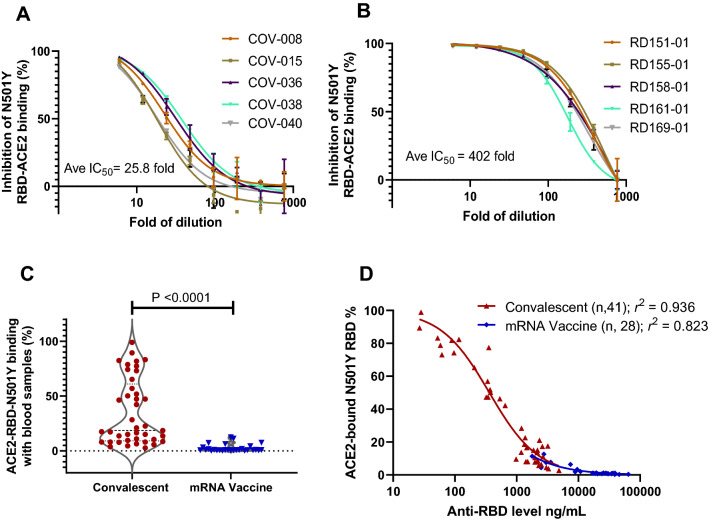
mRNA vaccine far more effective than natural immunity in neutralizing N501Y RBD mutant in ACE2 binding. (**A**) Dilution study to determine the IC_50_ of representative convalescent samples with median levels of anti-RBD antibodies. (**B**) Dilution study to determine the IC_50_ of representative vaccinated samples with median levels of anti-RBD antibodies. (**C**) Vaccinated blood samples more effective than convalescent ones to inhibit N501Y RBD in ACE2 binding (*P* < 0.0001). (**D**) Antibody concentration-dependent inhibition of N501Y RBD and ACE2 binding with blood samples from natural immunity (*r*^2^ = 0.926) and mRNA vaccination (*r*^2^ = 0.823).

To further determine the difference between natural immunity and mRNA vaccination, we selected five samples that had median levels of anti-RDB antibody of each group, and performed dilutions and neutralization studies against N501Y binding to ACE2. The results showed that dilution factors to IC_50_ were 25.8 and 402.0 for convalescent and mRNA vaccinated blood samples (Fig. 4A,B), a difference of 15.6-fold. This difference in neutralization is consistent with that the mRNA vaccinated blood had 16.8-fold higher anti-RBD antibody than the convalescent blood (Fig. 1B). Thus, the mRNA vaccinated blood is far more effective in neutralizing the high affinity N501Y RBD from binding to ACE2.

When tested for neutralizing N501Y RBD against ACE2 binding, the mRNA vaccinated blood was far more effective compared to convalescent samples to achieve minimum ACE2 binding (*P* < 0.0001; Fig. 4C). There were 31.7% convalescent and 0% of vaccinated samples retained over 50% N501Y RBD and ACE2 binding, a difference that is highly significant. Detailed examination of antibody concentration dependent neutralization revealed strong correlations between RBD antibody levels and neutralization activities for both sample groups (*r*^*2*^ = 0.94 and *r*^*2*^ = 0.82; Fig. 4D). Thus, the substantially elevated antibody levels in the mRNA vaccine group appeared to be the primary driver of better neutralization activities against the high affinity N501Y RBD. Similarly, improved efficacy with the vaccinated blood was also observed against the WT RBD and ACE2 binding (Supplementary Fig. 15). Therefore, the data indicates the insufficiencies of natural immunity, and the superiority of mRNA vaccination in neutralizing RBD and ACE2 binding, particularly against SARS-CoV-2 variants with increased affinity to their cell receptor.

## Discussion

In this study, we showed that mRNA vaccinated blood donors have a median of 17 times higher RBD antibody levels when compared with those who became seropositive due to prior COVID-19. Our results indicated an exceptional strong association between high RBD antibody levels in and the ability to biochemically neutralize RBD binding to the cellular ACE2 receptor. The N501Y mutation, while did not alter the neutralizing antibody binding, presented with a fivefold greater affinity to ACE2, which resulted in a drastically reduced ability of COVID-19 convalescent antisera to neutralize its ACE2 binding. Fortunately, the vaccinated blood samples, due to their much-elevated RBD antibody levels, were far more effective in neutralizing both the WT and N501Y RBD from binding to ACE. With an average of 16-fold greater potency than convalescent blood, the vaccinated blood samples were more than sufficient to compensate for the fivefold increased affinity of N501Y RBD, resulting in the highly effective inhibition of both the WT and N501Y RBD from binding to ACE2.

We observed very strong correlation between RBD antibody levels and ability to biochemically neutralize RBD and ACE2 binding. Previous studies have shown the correlation between neutralizing antibody and protection^34,35^. With over 150 million people infected with SARS-CoV-2 by May 2021, one of the critical questions going forward is whether the natural immunity would be sufficient to prevent future reinfections, particularly by more infectious variants. N501Y RBD is central to the investigation as it is the key driver to increased affinity to cell ACE2 receptors. While the reinfections were seen with the original SARS-CoV-2, our results indicated that the antisera from natural immunity would be less effective against variants such as B.1.1.7 due to its increased affinity to ACE2. Thus, many individuals acquired immunity through prior SARS-CoV-2 infections would not be sufficient to prevent reinfections by new variants with higher affinity to their cell receptors, especially in those with low RBD antibody levels.

Our findings would bring advances in the understanding of different vaccines and their abilities to fight off different SARS-CoV-2 variants. There are multiple vaccines tested in various geographic region with different prevalence of SARS-CoV-2 variants. In one study, mRNA vaccine BNT162b2 exhibited 97% efficacy against symptomatic COVID-19 in Israel dominated by B.1.1.7 variant^36^. Our study showed that not only the mRNA vaccinated plasma has 17-fold higher antibodies than the convalescent antisera, but also 16 time more potential in neutralizing RBD and ACE2 binding of both the original and N501Y mutation that was present in the above studies. Thus, the increased antibody levels were sufficient to compensate for the increase virulence due to higher ACE2 binding of this variant. In another study, an adenovirus-based vaccine ChAdOx1 nCoV-19 exhibited 55% clinical activity in the UK^16^ where B.1.1.7 was prevalent. A plausible explanation would be that this vaccine^37^ and another adenovirus vaccine^38^ only produced antibody levels that were comparable to COVID-19 convalescent blood. Thus, the clinical experience with B.1.1.7 suggests that higher levels of RBD antibody would be required to protect the subjects from infections. In the third example, a nanoparticle vaccine NVX-CoV2373 was only 50% effective in South Africa dominated by B.1.351^17^. The NVX-CoV2373 was be able to induce a fourfold increase over convalescent sera^39^. Thought the vaccination works against the difficult B.1.351 variant, there might be a room for further improvement by achieving higher antibody levels.

Similar to several previous reports on the durability of humoral response in people recovered from SARS-CoV-2 infection^40, 41^, there was no trend of decreasing RBD antibodies in those with natural immunity for up to 9 months in our dataset. However, our data revealed a large variation of their levels that were stable in given individuals. In comparison, while mRNA vaccines resulted in much higher RBD antibody levels than natural infections, this hyper-elevated level appeared to be less stable with samples at 6 months past the second dose. While our work is still very preliminary, there is a recent study observing similar rapid decline in RBD antibody within 6 months of BNT162b2 vaccine^42^, which is further strengthened by its clinical waning protection against SARS-CoV-2 infection in several studies^42–44^. It would be interesting to test more cases and over longer duration to see how fast the antibody levels would decline over time, particular in those with hyper-elevated antibody levels.

Likely many other surrogate biomarkers for a medical intervention on a disease, our in vitro receptor-binding neutralization has its limitations. There are factors that cannot be represented in this model. These include the 3D structures of the viral spike protein and ACE2, the surface density of both molecules, the process of viral entry into the cells and more. In addition, the mutation profiles of the variants are drastically simplified in our model. Other RBD mutations, K417N/T and E484K, either alone or in combinations, have not been evaluated in this study. Moreover, the IC_50_ values that we obtained with in vitro RBD-ACE binding assay, while biochemically informative, is not equivalent to an in vivo protective level. Besides, the quantification of RBD antibody levels of antisera is based on the calibration of a monoclonal antibody for consistency and universal adaptability, where the polyclonal nature of the antisera may be simplified. However, the RBD and ACE2 binding is perhaps the most crucial step for early viral pathogenesis, and the RBD variants are of the most concerns for current global health. Our data showed that the protein biochemical neutralization assay is a sensitivity, specificity, quantitative, and reliable biomarker to determine the neutralizing ability of antisera against SARS-CoV-2 and its variants. The biochemical neutralization assay has some advantages over viral or pseudo-viral based neutralization, as it is both highly sensitive and quantitative, and can be performed rapidly without cell culture, suitable to be applied in clinical settings. It can complement RBD serology assay to provide functional data, such as the binding affinity change of a new SARS-CoV-2 variant; or altered ability of anti-sera to neutralize a different variant. It could be used immediately as a surrogate for the clinical investigations of SARS-CoV-2 variants and the protection either by natural immunity or vaccination.

## Methods

### Patient or blood donor samples

Serum samples were collected at convalescent donors with prior documented COVID-19 under the NIH clinical protocol (ClinicalTrials.gov identifier: NCT04360278). A total of 41 samples were prospectively collected from 33 donors who had confirmed COVID-19 (Supplementary Table 1). An additional were collected under the same protocol from 28 people who never had COVID-19 but completed two doses of COVID-19 mRNA vaccines, either Pfizer or Moderna (Supplementary Table 2). All these samples were and processed at a single site within 4 h of blood draw in compliance of Good Clinical Practice. The samples were collected using the Institutional Review Board-approved protocol at the US National Institutes of Health with informed consent. Informed consent was obtained from all study participants. All methods were performed in accordance with the relevant guidelines and regulations. Patient samples were de-identified and tested in an unbiased and blinded fashion.

Additional samples from 38 patients were obtained from Department of Laboratory Medicine of NIH as diagnostics samples. The samples were selected based on the positive diagnoses and no personal or medical information was made available at any time for this research.

### RBD protein expression and purification

Recombinant SARS-CoV-2 spike receptor-binding domain (RBD) proteins contained a tissue plasminogen activator (TPA) signal peptide followed by amino acids 318–529 of spike (wild type or N501Y mutant) with a HRV-3C protease site and a C-terminal His (8)-streptavidin binding peptide tag. Proteins were expressed in Expi293 cells (ThermoFisher) for 96 h at 32 °C and purified as previously described^33, 45^. Final proteins were validated by mass spectrometry and size exclusion chromatography.

### RBD antibody assay

The recombinant RBD proteins were labeled with either biotin or MDS ruthenium-tag as previous described^46^. The RBD monoclonal antibodies for calibration (D003) and reference standard (D001) were acquired from Sino Biological (Wayne, PA, USA). For serology assay to determine the antibody levels of human sera, 2.5 µL of serum samples were diluted with diluent to 25 µL, mixed with 25 µL of 1 µg/mL biotin-RBD (capture) and 25 µL of 1 µg/mL ruthenium-RBD (detection). All reagents were added together into 96-well streptavidin-coated assay plates (Meso-Scale Diagnostics, Rockville, MD, USA). Both calibrator and reference standard were added in the place of sera. Incubation was carried out at room temperature for 1 h with constant shaking. After three washes with 150 µL of wash buffer, 150 µL of 2× read buffer was added, followed by reading with a Meso-Scale QuickPlex SQ120 within 5 min.

### Neutralization assay

The RBD proteins were labeled with MDS ruthenium-tag as described above. The recombinant human ACE2 (aa 18–740) was expressed in NS0 cells, affinity purified, and biotinylated (R&D Systems, Minneapolis, MN, USA). The streptavidin-coated was used for the assay, on which 25 µL of 1 µg/mL biotin-ACE2 were added and incubated at room temperature for 1 h. At the same time, serum neutralization was carried out with 60 ng/mL ruthenium-tag RBD mixed with 5 µL of human sera in a total of 30 µL (1:6 dilution of sera) and incubated at 37 °C for 1 h. They were then added to the biotin-ACE2 assay plate for a further 1 h incubation. This is followed by the addition of read buffer and QuickPlex reading. Standard calibration curves were obtained with diluted ruthenium-tag RBD starting from 200 ng/mL at the highest level without incubating with sera.

### Statistical analyses

Data analyses were performed using GraphPad Prism 8. Mann–Whitney non-parametric t test was used to compare results of two unrelated groups such as the RBD antibody levels of diagnostic and convalescent samples. Paired t test was performed in case of multiple blood samples from the same convalescent donors over time, to evaluate the changes in antibody levels and in neutralization abilities.

Correlative analysis was performed to evaluate the association of medical parameters, such as time to disease onset, age of disease onset, duration, and sex, with the levels of antibody. It was also performed to determine the assay specificity in the serum neutralization assay between the COVID-19 antisera and the pre-COVID-19 controls. ROC analysis was performed to determine the sensitivity and specificity of neutralization assays against the WT and the N501Y RBD proteins.

Four parameter logistic regression model provided by Meso-Scale Discovery Workbench 4.0 was used to determine the concentrations of anti-RBD antibodies in sera. Linear regression analysis with four parameters was performed to determine the linearity of both antibody assay and neutralization assay. It is also used to demonstrate the linear relationship in the activities of antisera in recognizing and in neutralizing both the WT and the N501Y RBD. It was further used to determine the relative ratios of these two proteins in binding to ACE2 in the absence and presence of COVID-19 sera from convalescent donors or diagnostic patients.

A nonlinear regression model with variable slope and four parameters was used to curve fit inhibitory dose–response data to determine the IC_50_ values for the neutralization of RBD in ACE2 binding. As all samples were diluted sixfold prior to the test, the results in the text were adjusted accordingly from those in the figures.

## Supplementary Information

Supplementary Information.
