# Supplementary material for: mRNA vaccine-induced antibodies more effective than natural immunity in neutralizing SARS-CoV-2 and its high affinity variants
Source: Sci Rep. 2022 Feb 16;12:2628. doi: 10.1038/s41598-022-06629-2 (PMC8850441; doi:10.1038/s41598-022-06629-2)
Supplement: Supplementary file 1 — Supplementary Information. [file 41598_2022_6629_MOESM1_ESM.pdf]

Supplementary Information

mRNA vaccine-induced antibodies more effective than natural immunity in neutralizing SARS-CoV-2 and its high affinity variants

Authors: Yunkai Yu[1], Dominic Esposito[2], Zhigang Kang[1], Jianming Lu[3], Alan T Remaley[4], Valeria De Giorgi[5], Leonard N. Chen[5], Kamille West[5], Liang Cao[1*]

Affiliations:

[1]Genetics Branch, National Cancer Institute, Bethesda, MD, USA

[2]Protein Expression Laboratory, NCI RAS Initiative, Frederick National Laboratory for Cancer Research, Frederick, MD, USA

[3]Codex BioSolutions, Inc, Gaithersburg, MD, USA

[4]Lipoprotein Metabolism Section, National Heat, Lung, and Blood Institute, Bethesda, MD, USA

[5]Department of Transfusion Medicine, National Institutes of Health Clinical Center, Bethesda, MD, USA

Supplementary Tables

Supplementary Table 1. Clinical information for COVID-19 convalescent donors and SARS-CoV2 mRNA vaccinated donors. Abbreviations: IQR, interquartile range. NA, not applicable.

Supplementary Table 2. Detail clinical information for COVID-19 mRNA vaccinated donors.

Supplementary Figures

Supplementary Fig. 1. Development of calibrator and reference standard for anti-RBD antibody test. (**A**) Mab D001 and D003 are mouse monoclonal antibodies against RBD. Lum units are direct luminescence reading from light signal from CCD camera. Mab D003 was subsequently

used as the calibrator and Mab D003 as the reference standard for quality assurance. Curve fitting was performed using sigmoidal 4PL model with X as concentration. (**B**) A results of assay optimization where the three-step assay was converted into a one-step assay where the capture RBD, the calibrator antibody D003, and the detection RBD were added simultaneously, followed by a single incubation before reading the results. Curve fitting was performed using sigmoidal 4PL model with X as concentration.

Supplementary Fig. 2. RBD serology assay validation data. (**A**) An example run to demonstrate the lower limit of sensitivity (LLoQ) and linear range of the quantification. (**B)** Receiver operating characteristic (ROC) analysis of the RBD serology assay. The area under the ROC curve is 1.000, indicative of perfect sensitivity and specificity. This is due to high sensitivity of the assay in all convalescent sera and the lack of any quantifiable antibody from all pre-COVID-19 sera (N = 171)

Supplementary Fig. 3. Test Comparison between anti-RBD test (this study) and Ortho's anti-S1 test. Correlative analyses were performed to compare the data of our anti-RBD results and Ortho's anti-S1 test results for the 41 convalescent donor samples. Two negative samples of Ortho's test were the 2nd samples following positive 1st tests. All samples were positive in our anti-RBD test. Both correlative analysis (r = 0.679, P < 0.0001) and linear regression analysis (r^2=0.461) were performed. Curve fit is based on simple linear regression model where the two sets of antibody data, while highly correlative, is not linear.

Supplementary Fig. 4. RBD antibody levels and age association. (**A**) Lack of age-RBD antibody level association in convalescent samples (*P* = 0.293). (**B**) Lack of age-RBD antibody level association in mRNA vaccinated samples (*P* = 0.361).

Supplementary Fig. 5. Rapid decline of RBD antibody levels in mRNA vaccinated individuals. T test of RBD antibody levels at indicated time points from those who completed mRNA vaccination (*P* = 0.0012).

Supplementary Fig. 6. The results of eight convalescent donors at two time points without a change in RBD antibody level (*P* = 0.396). The RBD antibody levels in given individuals are stable with a correlative coefficient *r* = 0.917 (*P* = 0.0007).

Supplementary Fig. 7. Sensitivity and specificity of neutralization assay against RBD-ACE2 binding with convalescent sera and pre-COVID-19 donor sera. The ROC analysis revealed an AUC = 0.986, indicative of an excellent performance of the neutralization assay.

Supplementary Fig. 8. Consistent neutralization activity over time against RBD-ACE2 binding with pair serum samples from convalescent donors. The correlative coefficient for the paring is effective with *r* = 0.952 (*P* = 0.0001).

Supplementary Fig. 9. Serum neutralization assay results using the second set of confirmatory COVID-19 diagnostic sera to verify the correlation between anti-RBD levels and neutralizing activity ($r^2 = 0.734$, $P < 0.0001$, N = 38).

Supplementary Fig. 10. Diagrams of RBD antibody quantification for both WT and N501Y RBD. The WT RBD was used to capture specific antibody in the sera, which were then detected with either the WT or N501Y RBD detecting agents.

Supplementary Fig. 11. Sensitivity and specificity of N501Y-RBD neutralization assay with convalescent sera shown with ROC analysis with the AUC = 0.948.

Supplementary Fig. 12. Neutralization assay results showing that the convalescent sera have similar neutralization activities against both WT and N501Y RBD with a slope of 1.032 using linear regression analysis (r^2 = 0.896, n = 41).

Supplementary Fig. 13. Linear regression analysis of the ACE2 bound WT and N501Y RBD without sera. The average ratio of ACE-2 bound N501Y over WT RBD is 5.1 (n = 5; slope range, 4.1 to 6.1). A representative of five consecutive experiments is shown.

Supplementary Fig. 14. Linear regression analysis of the ACE2 bound WT and N501Y RBD detected using the second set of discovery COVID-19 diagnostic sera. After neutralization, the ratio (slope) of ACE2-bound N501Y and WT RBD was 6.45 (r^2 = 0.810, n = 38).

Supplementary Fig. 15. Relative WT RBD and ACE2 binding in the presence blood samples from natural immunity and mRNA vaccination. (**A**) Drastic difference in the ability to inhibit N501Y RBD mutant in ACE2 binding between convalescent and vaccinated blood samples ($P <$ 0.0001). All blood samples were diluted 1:6 before the addition to N501Y RBD and protein

neutralization assay. (**B**) Diagram of correlations between relative N501Y RBD and ACE2 binding and anti-RBD antibody concentrations of blood samples from natural immunity and mRNA vaccination. The r^2 of both sample sets were shown.

Clinical information for convalescent and mRNA vaccinated donors

Characteristic	COVID-19 (N=33)	mRNA vaccine (N=28)	*P*
Median age (IQR) Yr	59.0 (45.0-64.0)	35.5 (24.0-49.0)	<0.0001
Sex No (%)			
Male	11 (33.3)	14 (50.0)	0.205
Female	22 (67.7)	14 (50.0)	
Median time from symptom (IQR) days	201.0 (175.5-232.5)	NA	
Median duration of disease (IQR) days	15 (10.0-21.0)	NA	
Median time from 2nd dose (IQR) days	NA	35.5 (30.3-41.0)	

Abbreviations: IQR, Interquartile range; NA, not applicable

Supplementary Table 1

Clinical information for COVID-19 mRNA vaccinated blood donor samples

Sample	Patient #	Vaccine Type	Dose #1	Dose #2	Date of Sample	Date post 2nd dose	Anti-RBD (ng/mL)
RD087-03	1	MODERNA	1/6/2021	2/6/2021	2/25/2021	19	64,551
RD133-07	2	MODERNA	1/22/2021	2/19/2021	3/11/2021	20	7,466
RD140-01	3	MODERNA	1/2/2021	1/30/2021	3/1/2021	30	17,684
RD141-01	4	MODERNA	12/29/2020	1/26/2021	3/2/2021	35	8,864
RD142-01	5	MODERNA	12/30/2020	1/25/2021	3/2/2021	36	39,518
RD143-01	6	MODERNA	8/6/2020	9/3/2020	3/3/2021	181	2,497
RD144-01	7	MODERNA	12/28/2020	1/25/2021	3/3/2021	37	39,124
RD145-01	8	MODERNA	1/4/2021	2/2/2021	3/4/2021	30	19,491
RD147-01	9	MODERNA	1/4/2021	1/29/2021	3/4/2021	34	10,815
RD149-01	10	PFIZER	1/11/2021	1/29/2021	3/8/2021	38	9,567
RD151-01	11	MODERNA	1/4/2021	2/2/2021	3/9/2021	35	27,842
RD153-01	12	MODERNA	1/22/2021	2/19/2021	3/9/2021	18	36,181
RD154-01	13	MODERNA	1/5/2021	2/3/2021	3/9/2021	34	45,759
RD155-01	14	MODERNA	1/20/2021	2/17/2021	3/10/2021	21	30,538
RD156-01	15	MODERNA	1/5/2021	2/2/2021	3/10/2021	36	17,063
RD157-01	16	MODERNA	1/6/2021	2/4/2021	3/10/2021	34	3,572
RD158-01	17	MODERNA	1/9/2021	2/7/2021	3/10/2021	31	28,500
RD159-01	18	MODERNA	1/4/2021	2/2/2021	3/15/2021	41	24,094
RD160-01	19	MODERNA	1/3/2021	2/2/2021	3/15/2021	41	34,185
RD161-01	20	MODERNA	1/5/2021	2/2/2021	3/15/2021	41	29,279
RD162-01	21	PFIZER	12/30/2020	1/20/2021	3/16/2021	55	2,766
RD164-01	22	MODERNA	8/17/2020	9/16/2020	3/16/2021	181	3,003
RD165-01	23	MODERNA	1/6/2021	2/3/2021	3/17/2021	42	20,542
RD166-01	24	PFIZER	2/4/2021	3/2/2021	3/17/2021	15	39,002
RD167-01	25	MODERNA	1/15/2021	2/12/2021	3/17/2021	33	34,631
RD168-01	26	MODERNA	1/9/2021	2/6/2021	3/17/2021	39	19,002
RD169-01	27	MODERNA	1/9/2021	2/6/2021	3/18/2021	40	25,509
RD170-01	28	PFIZER	8/28/2020	9/21/2020	3/18/2021	178	1,789

Supplementary Table 2

Development of calibrator and reference standard for anti-RBD antibody test (A), and assay optimization – conversion from three-step assay to one-step (B)

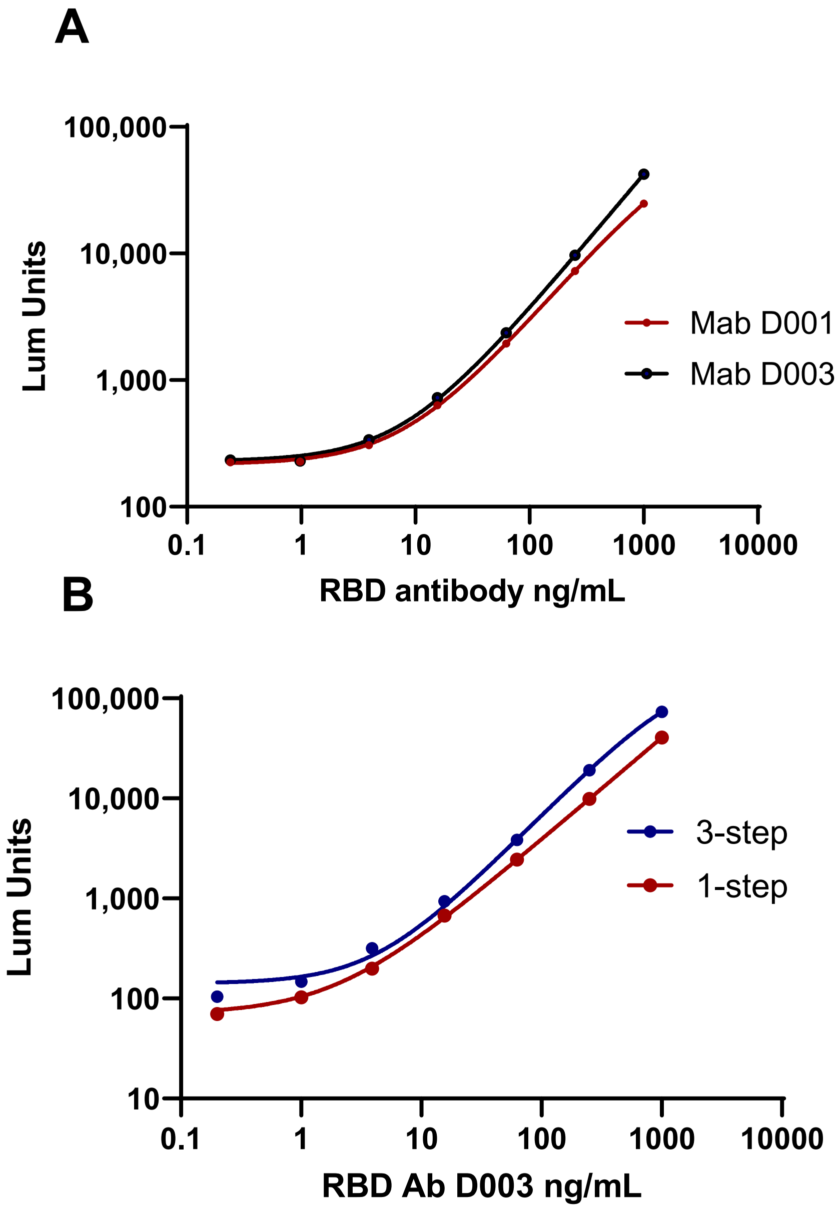

Supplementary Fig 1

COVID-19 RBD serology assay validation data

A

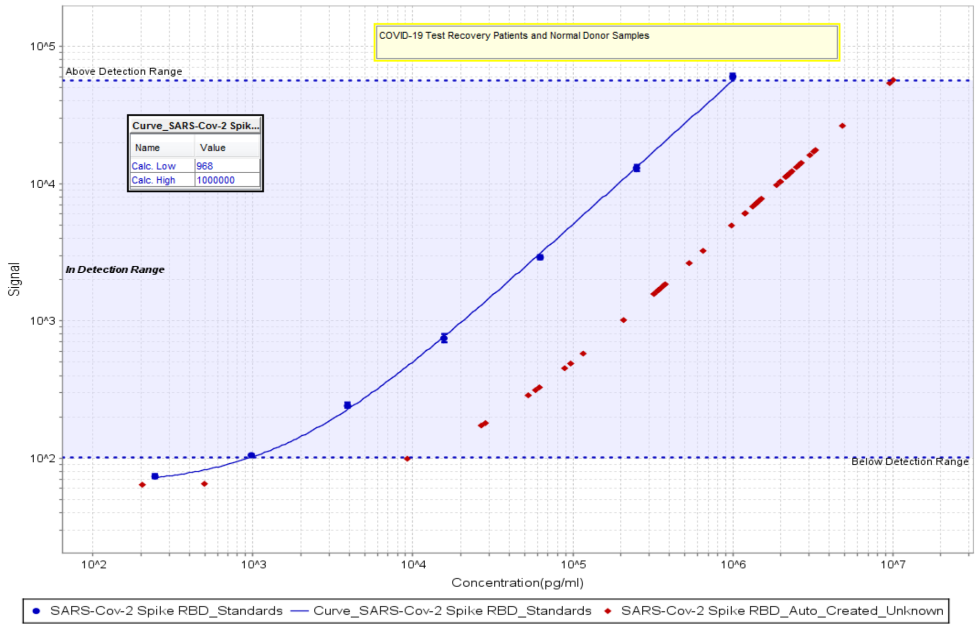

B

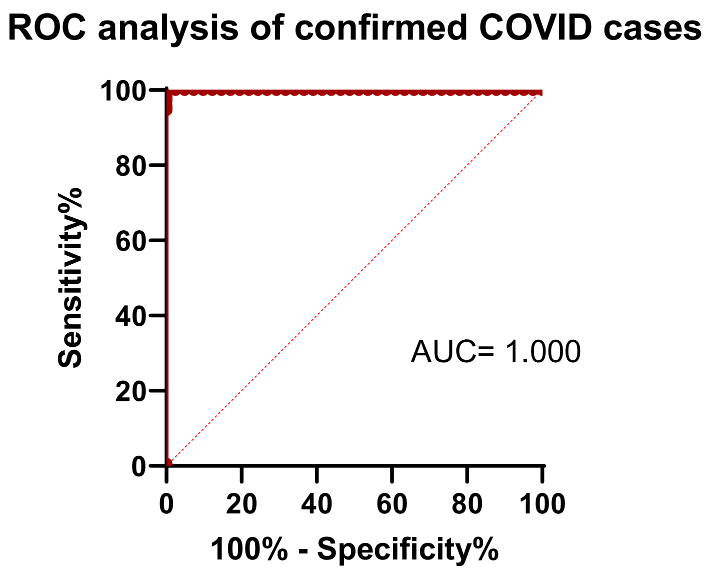

Supplementary Fig 2

Test Comparison between anti-RBD test (this study) and Ortho's anti-S1 test

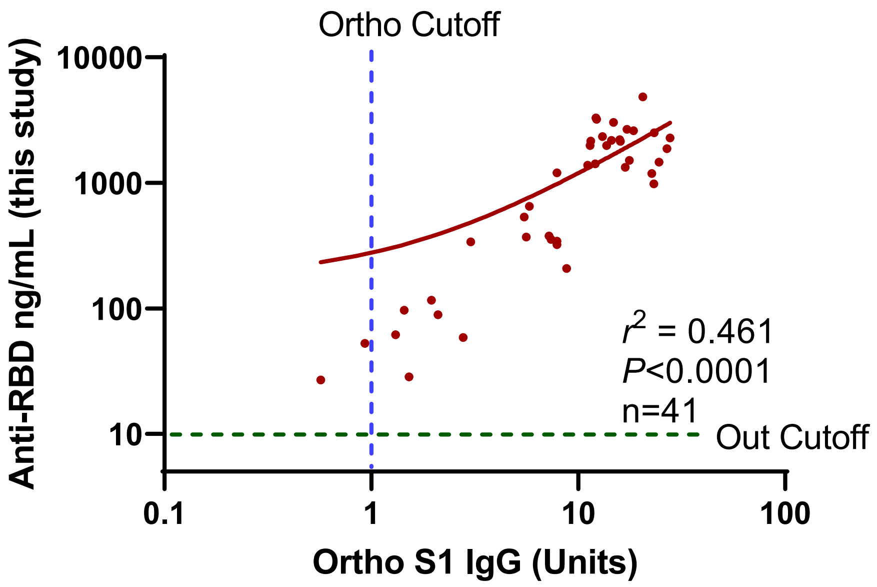

Correlation bet anti-RBD and Ortho tests

Pearson r	
r	0.6787
95% confidence interval	0.4690 to 0.8160
R squared	0.4607

Supplementary Fig 3

Anti-RBD levels and age correlation for both COVID-19 convalescent and mRNA vaccinated donors

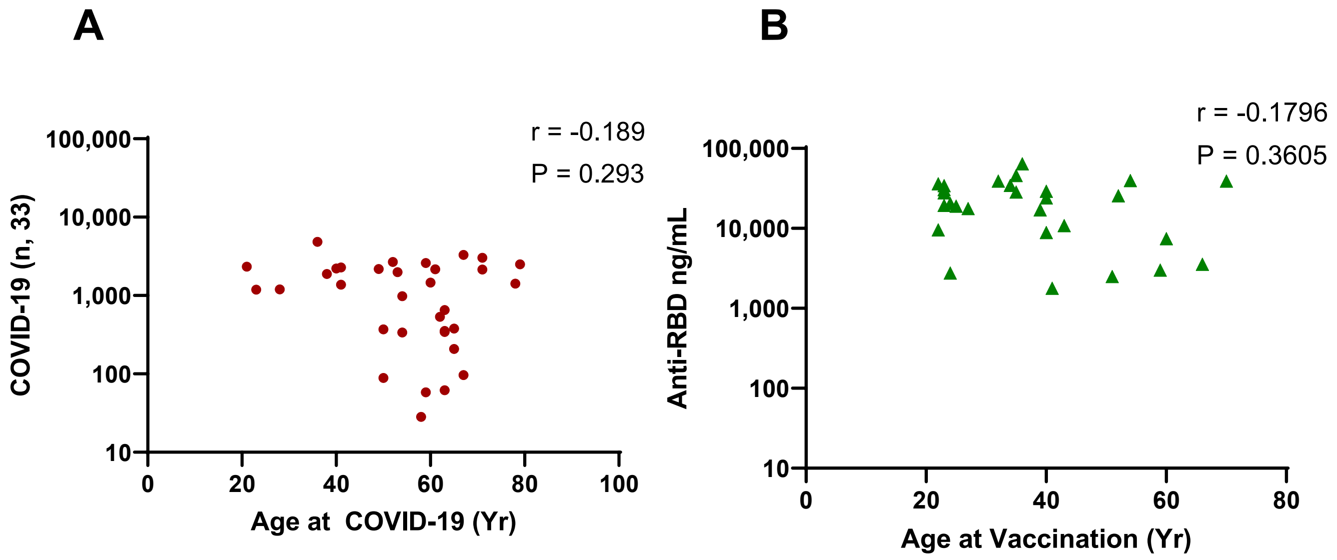

Supplementary Fig 4

Reduced RBD antibody levels at 6 mo post mRNA vaccination

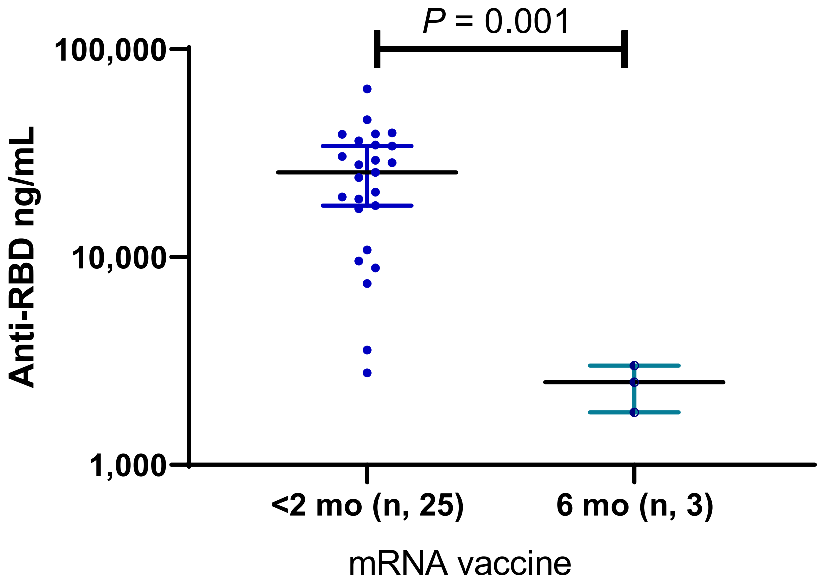

Supplementary Fig 5

Relative stability of RBD antibody at time points post COVID-19

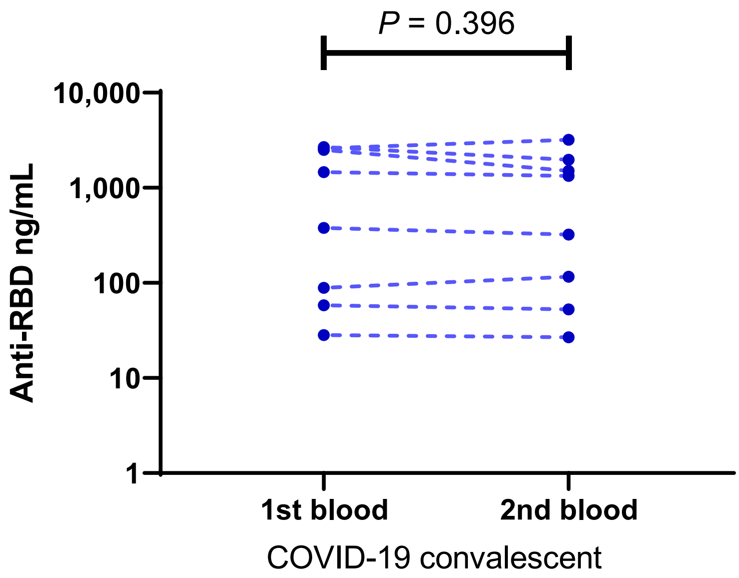

Supplementary Fig 6

Clinical performance of antibody RBD neutralization assay using Receiver Operator Characteristic (ROC) analysis

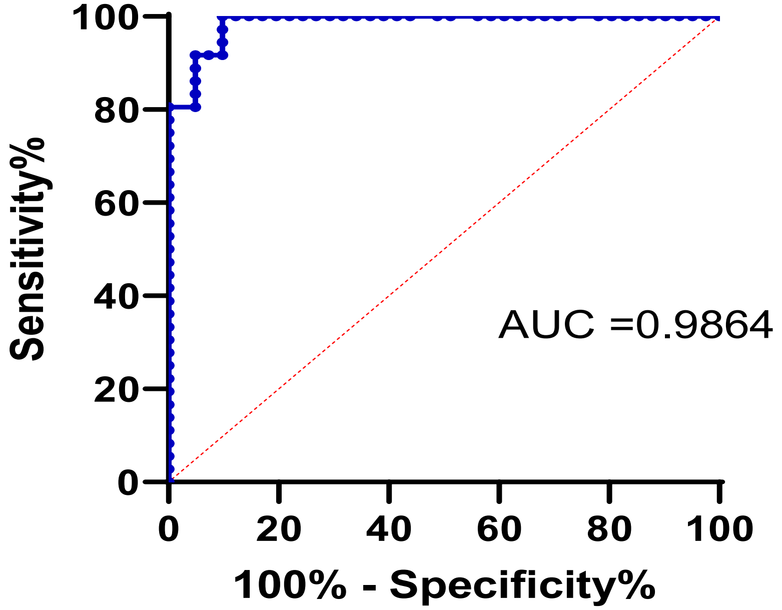

Supplementary Fig 7

Consistency of RBD-ACE2 binding neutralization assay with paired convalescent serum samples taken at different time points

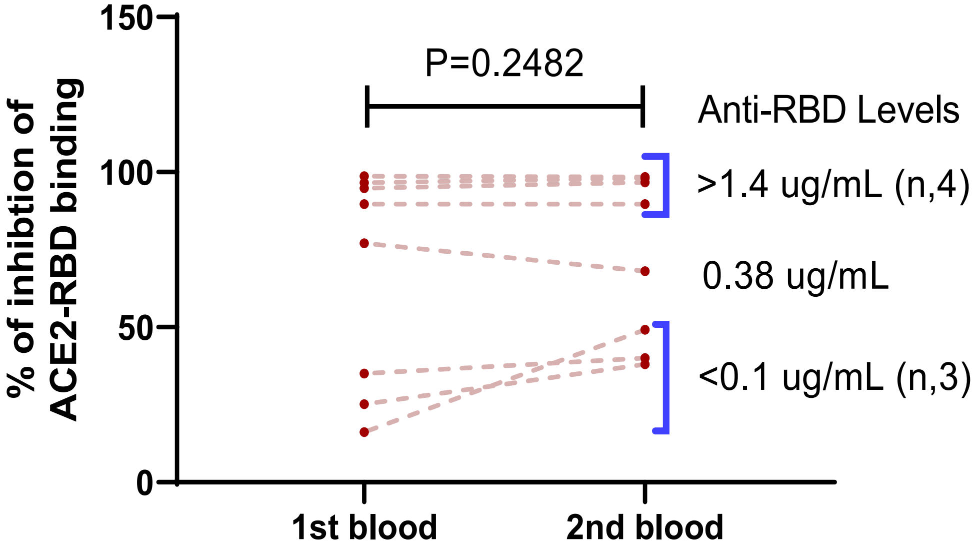

Correlation coefficient (r)	0.9520
P value (one tailed)	0.0001

Supplementary Fig 8

Serum neutralization assay results using the second set of confirmatory COVID-19 diagnostic sera

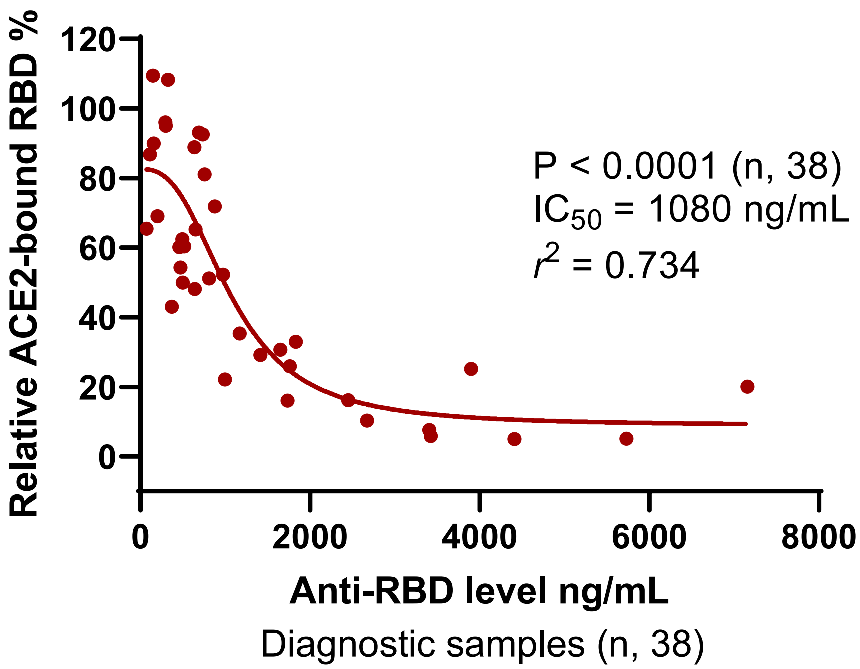

Supplementary Fig 9

Diagram on immunoassays detecting antibodies levels against both WT and N501

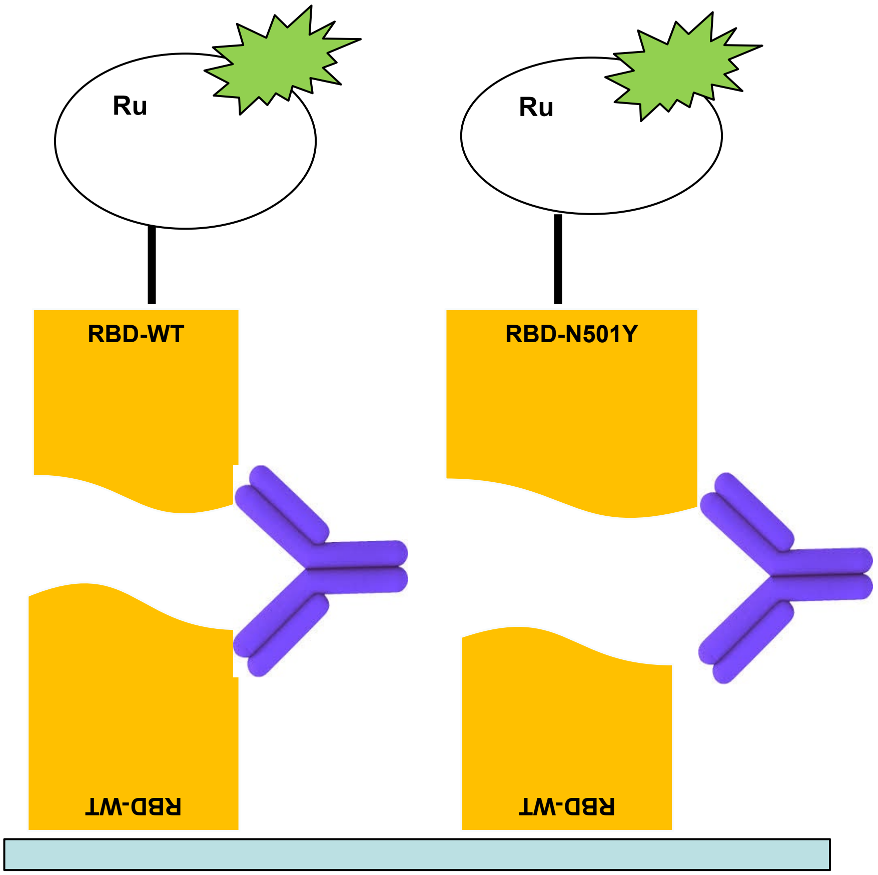

Supplementary Fig 10

Determination of the sensitivity and specificity of serum neutralization assay against the N501 RBD

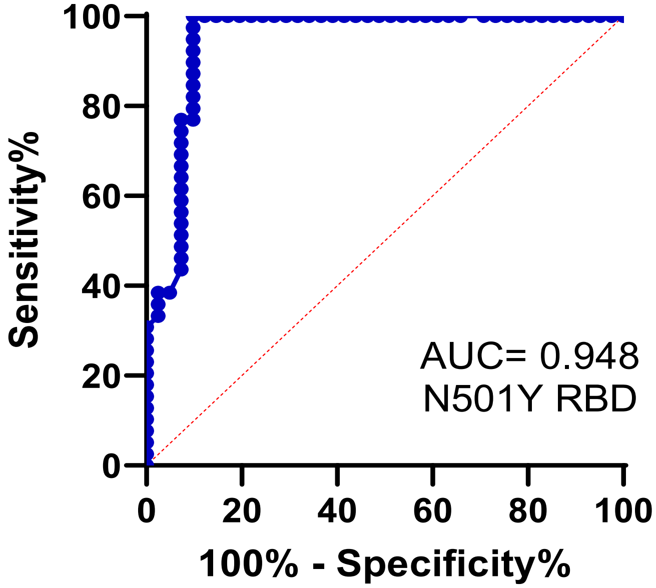

Supplementary Fig 11

Comparison of convalescent sera in neutralizing the WT and N501Y RBD against ACE2 binding

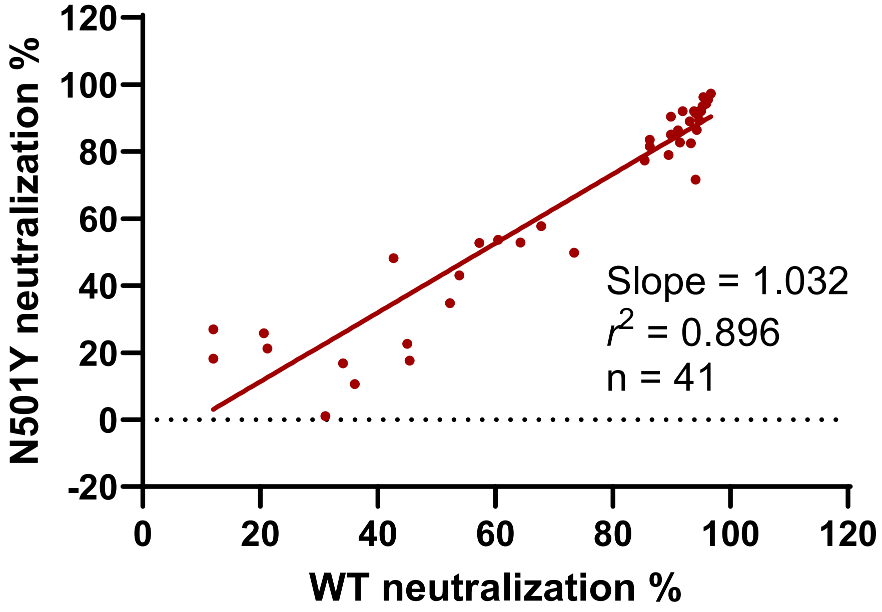

Supplementary Fig 12

N501Y-RBD has 5-fold increase in ACE2 binding compared with the WT RBD

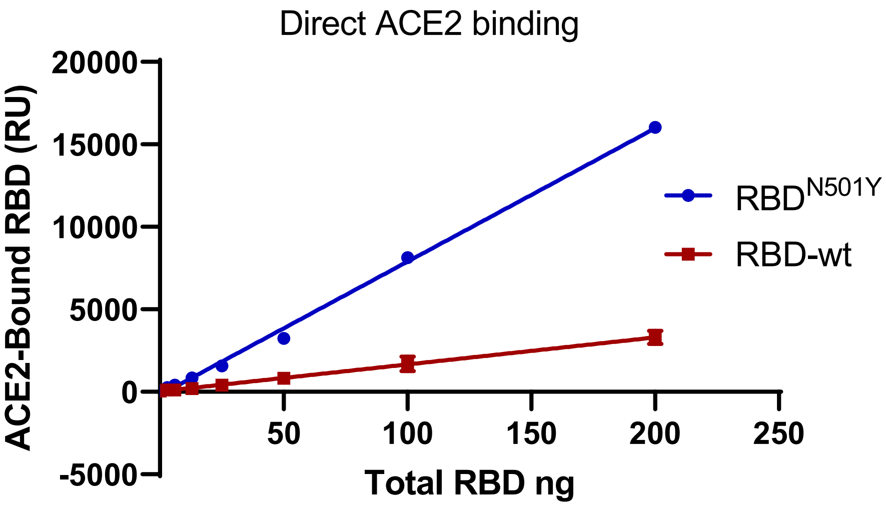

Summary	RBD-wildtype	RBD-N501Y
Ave R^2	0.969	0.988
Ave Slope Ratio	1.00	5.14
No of Repeats	5	5

Supplementary Fig 13

COVID-19 diagnostics sera and their neutralization activities against the WT and N501 RBD

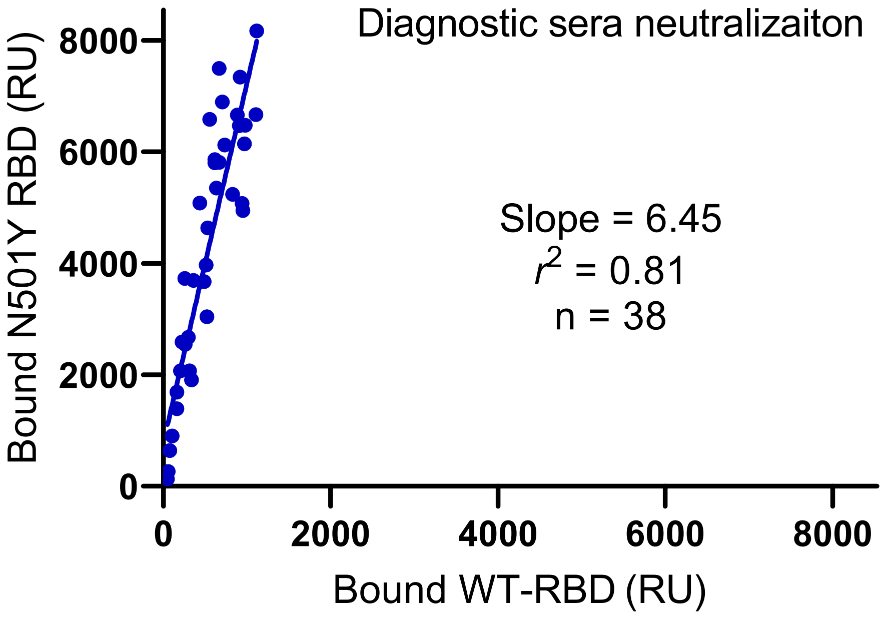

Supplementary Fig 14

mRNA vaccine blood is far more effective in neutralizing the WT RBD and ACE2 binding

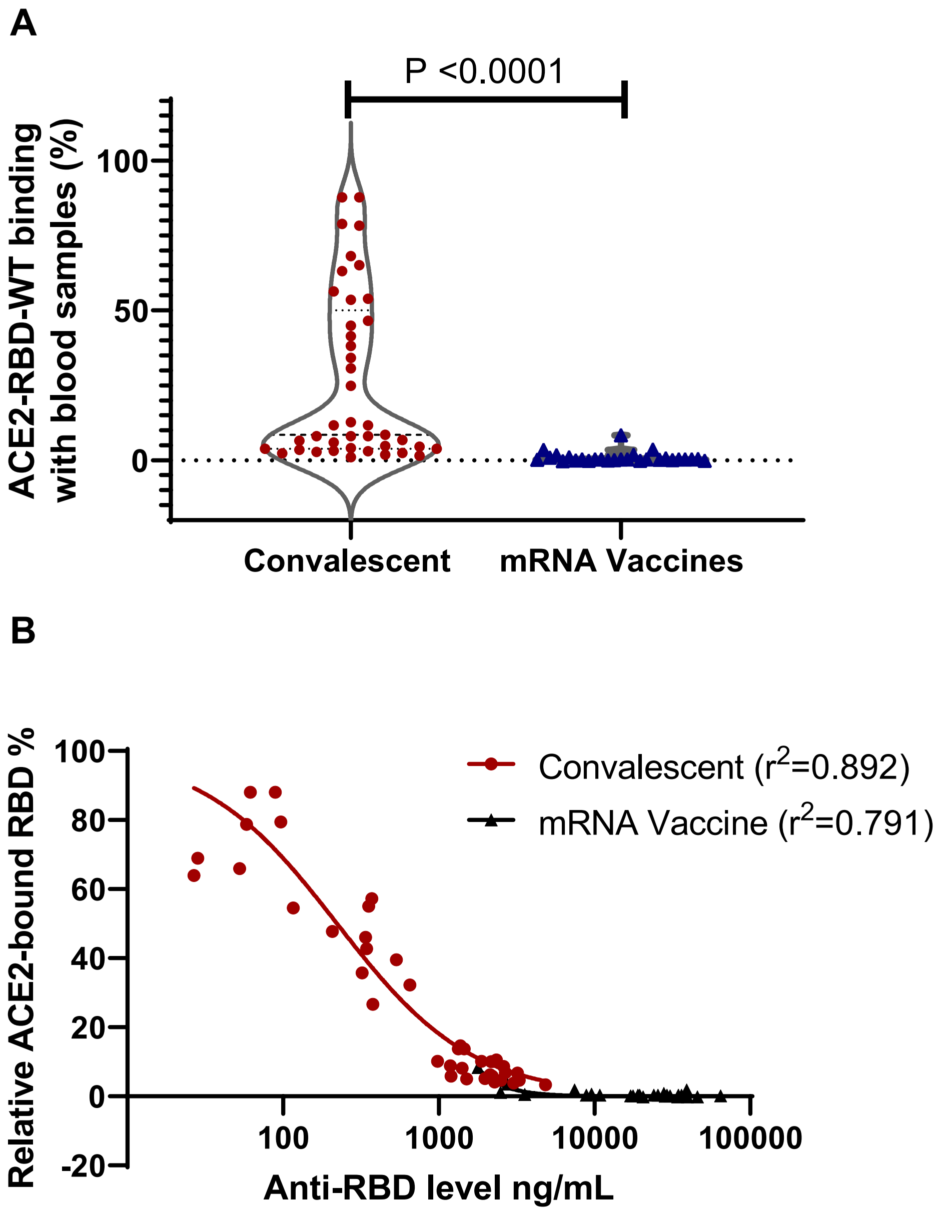

Supplementary Fig 15
